# Inner Nuclear Layer Thickening Is Inversley Proportional to Retinal Ganglion Cell Loss in Optic Neuritis

**DOI:** 10.1371/journal.pone.0078341

**Published:** 2013-10-03

**Authors:** Megha Kaushik, Chen Yu Wang, Michael H. Barnett, Raymond Garrick, John Parratt, Stuart L. Graham, Prema Sriram, Con Yiannikas, Alexandr Klistorner

**Affiliations:** 1 Save Sight Institute, University of Sydney, Sydney, New South Wales, Australia; 2 Brain and Mind Research Institute, University of Sydney, Sydney, New South Wales, Australia; 3 Department of Neurology, St Vincent’s Hospital, Sydney, New South Wales, Australia; 4 Department of Neurology, Royal North Shore Hospital, Sydney, New South Wales, Australia; 5 Ophthalmology and Vision Science, Macquarie University, Sydney, New South Wales, Australia; 6 Department of Neurology, Concord Hospital, Sydney, New South Wales, Australia; Univeristy of Melbourne, Australia

## Abstract

**Aim:**

To examine the relationship between retinal ganglion cell loss and changes in the inner nuclear layer (INL) in optic neuritis (ON).

**Methods:**

36 multiple sclerosis (MS) patients with a history of ON and 36 age and sex-matched controls underwent Optical Coherence Tomography. The paramacular retinal nerve fiber layer (RNFL), combined ganglion cell and inner plexiform layers (GCL/IPL) and inner nuclear layer (INL) thickness were measured at 36 points around the fovea. To remove inter-subject variability, the difference in thickness of each layer between the ON and fellow eye of each patient was calculated. A topographic analysis was conducted.

**Results:**

The INL of the ON patients was thicker than the controls (42.9µm versus 39.6µm, p=0.002). ON patients also had a thinner RNFL (27.8µm versus 32.2µm, p<0.001) and GCL/IPL (69.3µm versus 98.1µm, p<0.001). Among the controls, there was no correlation between RNFL and GCL/IPL as well as RNFL and INL, but a positive correlation was seen between GCL/IPL and INL (r=0.65, p<0.001). In the ON group, there was a positive correlation between RNFL and GCL/IPL (r=0.80, p<0.001) but a negative correlation between RNFL and INL (r=-0.61, p<0.001) as well as GCL/IPL and INL (r=-0.44, p=0.007). The negative correlation between GCL/IPL and INL strengthened in the ON group when inter-subject variability was removed (r=-0.75, p<0.001). Microcysts within the INL were present in 5 ON patients, mainly in the superior and infero-nasal paramacular regions. While patients with microcysts lay at the far end of the correlation curve between GCL/IPL and INL (i.e. larger INL and smaller GCL/IPL compared to other patients), their exclusion did not affect the correlation (r= -0.76, p<0.001).

**Conclusions:**

INL enlargement in MS-related ON is associated with the severity of GCL loss. This is a continuous relationship and patients with INL microcysts may represent the extreme end of the scale.

## Introduction

Multiple sclerosis (MS) is recognized as a chronic inflammatory demyelinating disease with concomitant neurodegeneration. Acute optic neuritis (ON) is a common feature of MS and is the first clinical manifestation in approximately 20% of MS patients [1]. In ON significant axonal loss occurs following the acute inflammatory process, which eventually results in retinal ganglion cell (RGC) neuronal loss through retrograde degeneration. This mechanism is now well established by numerous studies [2-6].

There are also reports of other retinal elements being affected in MS. Post-mortem studies, supported by electrophysiological investigations, have demonstrated significant pathological changes in retinal cells deeper to the retinal ganglion cell layer (GCL), including bipolar cells and photoreceptors [7-9].

More recently, high-resolution spectral-domain Optical Coherence Tomography (OCT) has created an opportunity to perform qualitative structural analyses of individual retinal layers *in vivo*. A number of OCT-based studies have revealed different abnormalities in the inner nuclear layer (INL) in eyes of MS patients. Some have reported disproportionate thinning of the INL [7,10], but to the contrary, a more recent study found an increased thickness of the INL in eyes of MS patients [11]. A small but not statistically significant increase of INL thickness in ON patients was also reported by Sriram et al (2012) [12].

In addition, several investigators have reported microcysts of the INL in various optic neuropathies and neurological diseases, including MS (with and without a history of ON). In MS, the presence of INL microcysts has been associated with disease severity [11]. Several mechanisms including autoimmune, inflammatory and mechanical factors have been suggested to explain the origin of these microcysts [11,13-16]. However, the question of the true nature of INL changes in MS still remains open.

One noteworthy problem is that most studies on the INL to date have been limited by the inability to accurately segment the INL from the outer plexiform layer, which can potentially mask true changes in the INL, reducing sensitivity. In addition, no relationship between RGC loss and the INL has been explored but we hypothesize that there is an association. Therefore, the objective of this study was to use spectral-domain OCT and a custom designed automated retinal layer segmentation algorithm to clarify structural changes in the INL in patients with MS-related ON and to assess the relationship between RGC loss and changes in the INL. We also investigated the prevalence and distribution of INL microcysts in MS-related ON.

## Materials and Methods

### Ethics Statement

Approval was obtained from the Human Research Ethics Committee of the University of Sydney (number 2012/744) and written informed consent was obtained from all participants.

### Participant selection

A cross-sectional retrospective study was completed on a total of 36 patients with clinically defined MS (based on the criteria of McDonald et al) and a history of acute ON as diagnosed by a neuro-ophthalmologist or neurologist [17].

Exclusion criteria included concomitant ocular or neurological disease or recurrent ON. Only MS patients with a history of ON of at least 3 months prior to participation in the study were included. Based on self-reporting and analysis of medical records, we documented the time of diagnosis of MS and ON as well as the duration between the last episode of ON and date of OCT for the study.

The MS patients were compared to 36 age and sex matched healthy controls who were selected from among the Save Sight Institute staff. These controls were included based on criteria of 6/6 vision, and no history of ocular disease, diabetes mellitus or high refractive error (outside of the range between ± 4 diopters). We also excluded MS patients with refractive error outside the range of ± 4 diopters.

### OCT Segmentation and Analysis

**Figure 1:**
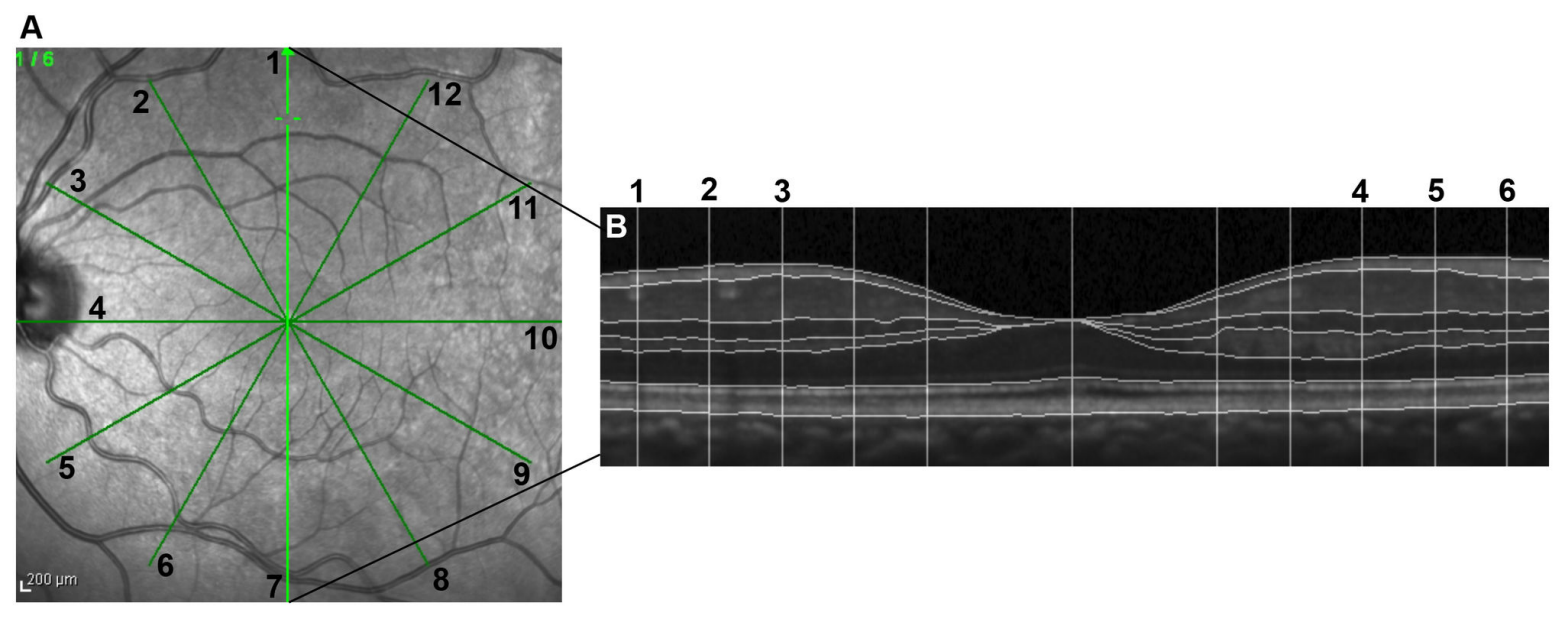
OCT segmentation pattern A. Fundus view: OCT segmentation along 12 radial lines through the central fovea. B. Cross-sectional view: Along each scan line, the thickness of each retinal layer was measured at 6 different points (3 points along each radial line). The 5 points closest to the fovea were not included in the analysis due to variability in automated retinal layer segmentation in this area as a result of indistinct layers.

All participants underwent OCT using Spectralis HRA+OCT (Heidelberg Engineering, Heidelberg, Germany). Only one eye was scanned in the control group which was chosen at random, but both eyes were scanned in the ON group. The macula radial pattern protocol was used, which provided 6 slices in a star-like pattern; the central fovea being the midpoint of each slice (Figure 1). This created 12 radial sections around the central fovea, each 4.5mm long and each separated by 30 degrees from each other. In total, 30 degrees of visual angle (15° of eccentricity) were scanned. Only scans that were of good quality, defined by signal strength greater than 25db, good centration of the scan, and uniform brightness were used. One hundred scans were averaged for each line scan to produce a resolution of 1536 pixels.

For each OCT slice around the fovea, segmentation of the retinal layers was performed automatically using a custom designed algorithm which applied vessel detection and removal, multiple size median filtering, and Canny edge detection to identify borders of the retinal layers. This program was used in a previous study conducted by our research group [12]. This program was used to measure the thickness of the paramacular retinal nerve fiber layer (RNFL), the combination of the ganglion cell and inner plexiform layers (GCL/IPL), and INL (Figure 1). The GCL and IPL could not be accurately distinguished due to similar reflectivity and thus the combined thickness of these two layers was used. Any differences in the GCL/IPL thickness between all eyes were attributed to the RGCs as the combined layers are thought to predominantly house the RGC neurons. The thickness of each layer was calculated at 3 separate points along each radial line. Given that there were 12 radial lines, a total of 36 points around the macula were measured. Only 3 points along each radial line were used for analysis as the layers are more distinct in this range.

We measured the average RNFL, GCL/IPL and INL thickness for each participant. We also measured the average thickness of the RNFL, GCL/IPL, and INL at each of the 36 points around the macula for both the controls and ON patients. To measure the deviation in RNFL, GCL/IPL and INL thickness in ON from normal values, we calculated the difference in average thickness of each layer between the control and ON groups at each of the 36 points across the retina. These calculations were topographically mapped. To minimize inter-subject variability we performed an asymmetry analysis by calculating the difference in the thickness of the retinal layers between the ON eye and the fellow eye for each subject.

In all participants we looked for microcysts in the retina, defined by well-circumscribed areas of hyporeflectivity.

### Statistics

Statistical analysis was conducted using Microsoft Excel, version 2007 (Microsoft, Redmond WA) and SPSS Statistics 21.0.0 (IBM, New York). The means, standard deviation (SD) of means, and Pearson’s correlation coefficient were calculated. Groups were compared using the student t-test and bivariate correlation. P-values less than 0.05 were considered statistically significant.

## Results

A total of 36 patients with a history of MS-related ON and 36 age and sex matched healthy controls were included in this study. The mean age was 39.5 for both controls (SD ± 10.7) and patients with ON (SD ± 9.8). The ratio of females to males was 28:8 among the ON patients compared to 27:9 for the control group.

All the patients with a history of ON were diagnosed with relapsing remitting MS (RRMS). All MS patients had a history of unilateral ON except for one who had binocular disease (however only one eye was included in the study for this patient). The average duration between the last episode of acute ON and OCT for this study was 51 months (range 3-203 months). The average duration of MS from diagnosis was 52 months (range 3-178 months). Three MS patients were on Fingolimod at the time of OCT testing.

### Correlations between the RNFL, GCL, and INL

For all participants we measured the thickness of the RNFL, GCL/IPL, and INL by averaging 36 points of measurement around the macula. Compared to normal controls the ON patients had a thinner RNFL (27.8µm ± 4 versus 32.2µm ± 2, p <0.001) as well as a considerably thinner GCL/IPL (69.3µm ± 14 versus 98.1µm ± 5, p <0.001). On the contrary, the average thickness of the retinal INL of patients with ON compared to the control group was significantly larger (42.9µm ± 6 versus 39.6µm ± 3, p= 0.002).

**Figure 2:**
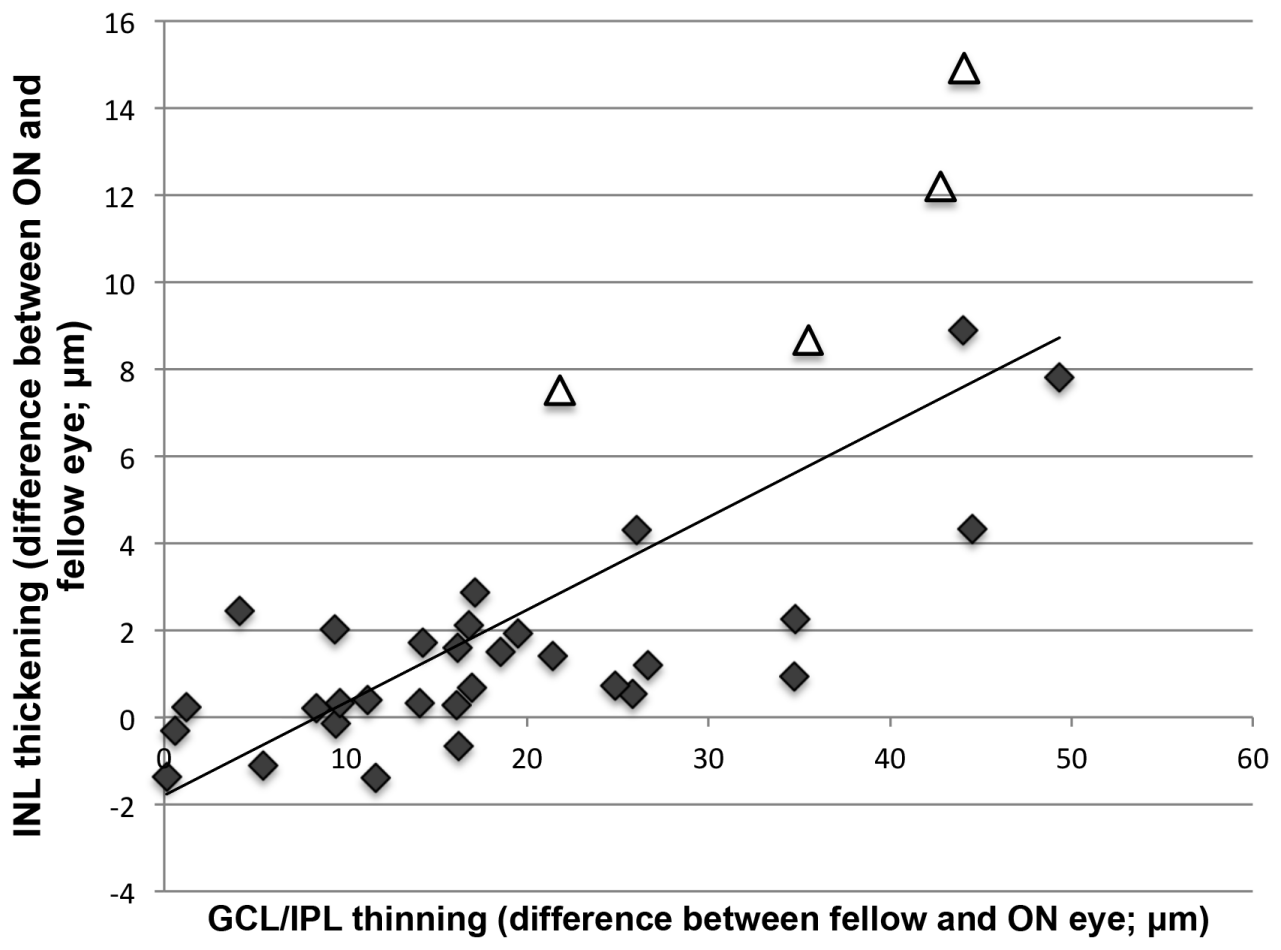
Correlation between GCL/IPL and INL in the ON group after removing inter-subject variability. The four triangles represent the ON patients with INL microcysts. The diamonds represent the ON patients without microcysts.

A closer examination revealed different relationships between the average GCL/IPL, RNFL, and INL thickness in the control and ON groups. Among the healthy participants, there was no association between the RNFL and INL (r= 0.03, p=0.85) as well as the RNFL and GCL/IPL (r= 0.14, p= 0.40), but there was a significant positive correlation between the GCL/IPL and INL thickness (r= 0.65, p<0.001). In contrast, in the ON group there was a strong positive correlation between the RNFL and GCL/IPL (r=0.80, p<0.001), which is expected since axonal degeneration is followed by neuronal loss due to retrograde degeneration. However, both the RNFL and the GCL/IPL demonstrated a highly significant negative correlation with the INL (r= -0.61, p<0.001 and r= -0.44, p=0.007 respectively). This inverse correlation between the GCL/IPL and INL (i.e. a thicker INL with a thinner GCL/IPL) strengthened in the ON group when asymmetry analysis was used (r= -0.75, p<0.001; see Figure 2).

There was no correlation between INL thickness and the duration between ON and OCT testing (r= -0.02, p=0.91) and the duration of MS (r= -0.02, p=0.93).

### INL Microcysts

In a total of 5 optic neuritis patients (14%), there was evidence of macular microcysts in the INL. The patient with a history of binocular ON had evidence of microcysts in the eye that was included in the study, but this patient was excluded from the asymmetry analysis. Eyes examined in the control group did not have evidence of INL microcysts. There was no significant difference in the average time between ON and OCT testing between patients with and without INL microcysts (62 ± 25 versus 49 ± 44 months respectively, p=0.60).

**Figure 3:**
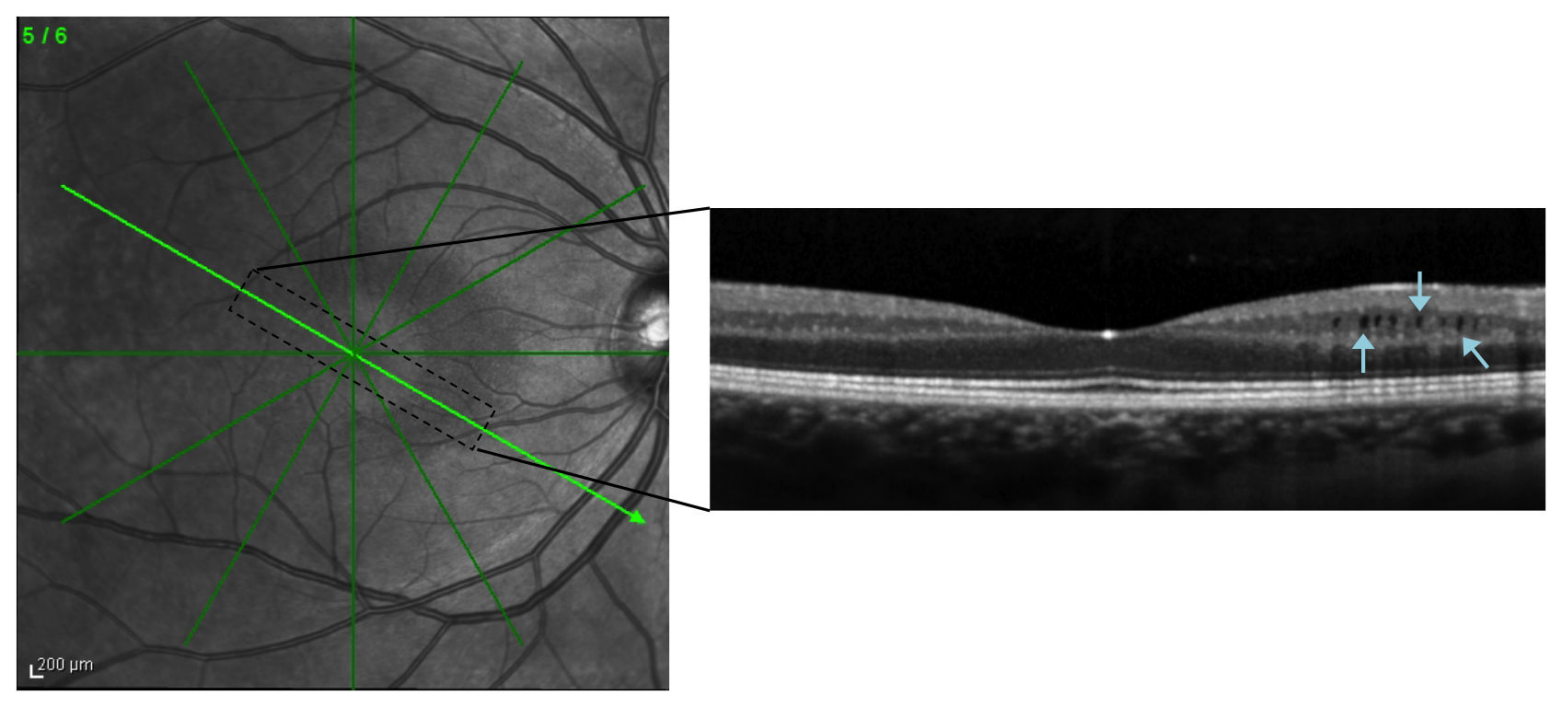
Microcysts in the INL of a patient with a history of acute ON. These microcysts appear more hypo-intense (indicated by the arrows) and are located in the nasal paramacular region but not temporally.

The morphology of the microcysts was noted to be elongated in shape perpendicular to the retinal layers (Figure 3). In all 5 eyes the microcysts were also more abundant and larger in the superior and inferior nasal quadrants of the macula, between 0.75mm to 2.0mm from the fovea along the 3^rd^, 4^th^ and 5^th^ radial lines (as numbered in Figure 1). These areas appeared distinctly more hypo-intense on the fundus OCT view. No microcysts were present temporal to the fovea.

The 5 eyes with microcysts had greater differences in the thickness of their retinal layers compared to ON eyes without microcysts. Thus, as expected, the INL was significantly larger in the patients with microcysts compared to those without microcysts (53.8µm ± 6 versus 41.2µm ± 3, p=0.007). The RNFL and GCL/IPL, however, were much thinner in patients with microcysts compared to those without (22.7µm ± 4 versus 28.6µm ± 4, p= 0.02; and 54.2µm ± 6 versus 71.7µm ± 14, p<0.001 respectively).

Figure 2 shows that on a correlational curve, the patients with INL microcysts concentrated at the higher end of the spectrum, with a greater increase in INL and decrease in GCL/IPL compared to other patients with ON. Since theoretically it is possible that the presence of microcysts (which increases INL thickness dramatically) is responsible for the observed correlation between the GCL/IPL and INL, we reanalyzed our data excluding eyes with microcysts. However, exclusion of these patients did not affect the overall correlation between the GCL/IPL and INL (r= -0.76, p<0.001). Also, whilst an increase in the average INL thickness in ON patients compared to normal controls became less prominent, it remained highly significant (ON eyes without microcysts: 41.0µm ± 2 versus controls: 39.8µm ± 2, p=0.004).

### Topographical relationship between changes in the RNFL, GCL/IPL and INL

**Figure 4:**
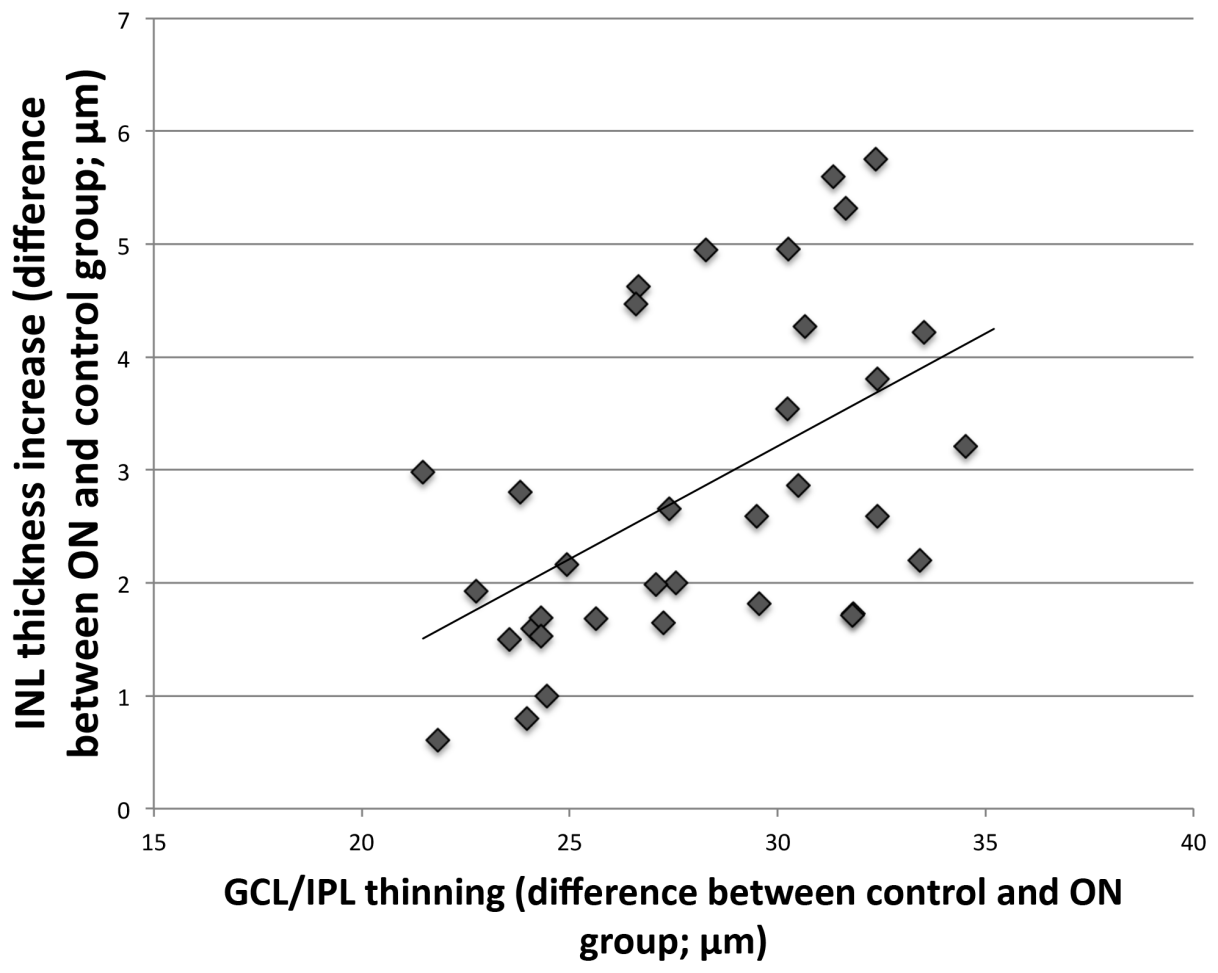
Correlation between GCL/IPL thinning and increase in the INL at different points across the paramacular region. This increase/decrease thickness at each point was calculated as the deviation in average layer thickness in the ON group from the normal, which is defined by the control group.

A comparison was made between the average thickness of the retinal layers in the ON group and the control group at individual points across the retina. As illustrated in Figure 4, we found an inverse correlation between the greater INL thickness and reduced GCL/IPL thickness in the ON group compared to the controls (r= -0.52, p=0.001). There was pronounced nasal-temporal asymmetry with the greatest thinning of RNFL and GCL/IPL and largest thickening of the INL seen between the optic disc and fovea compared to the temporal retina. The greatest thinning of the GCL/IPL and greatest increase in the INL thickness occurred supero-nasally and infero-nasally to the macula.

## Discussion

This study confirmed a significant increase in INL thickness in ON eyes of MS patients. In addition, it demonstrated that INL enlargement is inversely proportional to the loss of RGCs, which is also reflected in topographical correspondence between the two. Compared to the normal controls, the largest decrease in the GCL/IPL and RNFL and greatest INL increase were seen between the optic disc and fovea, whilst the opposite was true for the area temporal to the macula. We also confirmed a small prevalence of microcysts in the INL and identified that eyes with microcysts demonstrated extreme thinning of the GCL/IPL and RNFL. However, this subset of eyes with microcysts was not solely responsible for the association between loss of RGCs and increased thickness of the INL.

In the control group, a lack of correlation of RNFL thickness with GCL/IPL and INL thickness reflects retinal connectivity, as the neuronal fibers of the RNFL do not originate from RGCs and INL cells directly adjacent to them. Majority of the RNFL fibers that were measured on OCT around the macula were connected to RGCs further in the periphery. However, in the ON group, the relationship between RNFL and GCL/IPL thickness became positive due to the severity of damage. There was in contrast, a significant positive correlation between the thickness of the GCL/IPL and INL in the control group as the adjacent cells in both layers are directly connected. This correlation became negative in the ON group and became even stronger after adjusting for inter-subject variability. This suggests a strong association between loss of RGCs and INL enlargement. The inverse relationship between the thickness of the INL and GCL/IPL, which has not been described in the literature, indicates that damage of RGCs leads to structural changes within the retina, affecting other retinal layers.

Thinning of the innermost layers of the retina (RNFL and GCL) has been consistently reported in ON [6,18-20]. However more variability exists in reports on changes in the deeper retinal layers. While INL thinning has been documented in a post-mortem study [7] and in some *in vivo* OCT studies [2,10], others observed no significant changes in INL thickness in ON patients with MS and neuromyelitic optica [6,20]. Moreover, two studies have documented greater INL thickness via OCT segmentation in ON patients [5,11]. However, segmentation of the outer plexiform layer from the INL was not conducted in these studies. A previous study by our research group was able to isolate the INL from the outer plexiform layer and found a small increase in INL thickness in ON patients which was not statistically significant [12]. In retrospect, the lack of significance of this result may have related to the analysis of only vertical OCT slices of the retina as well as the inclusion of more peripheral points beyond the macula in the thickness analysis where the layers are less distinct. An earlier version of the segmentation algorithm was also not robust enough to provide a clear segmentation in all cases.

Our study revealed INL microcysts in a small proportion of patients with ON. Other studies similarly report a small prevalence of INL microcysts ranging from 0.8 to 6% [11,13,15]. These patients had more severe thinning of the RNFL and RGC/IPL, which is again consistent with other studies [13]. However, not all patients in our study with severe RNFL and RGC loss had microcysts in the INL, which may suggest individual susceptibility.

The presence of microcysts in the INL in patients with ON has recently attracted significant attention as a possible marker of MS activity. It was reported that these patients have worse vision, more severe disease and greater disability [11,13]. It has even been suggested that they may represent a unique and severe phenotype of MS [11]. Our results suggest that INL enlargement in eyes of MS patients represents a continuous and strongly dependent relationship with the integrity of RGCs with microcysts representing the extreme end of the spectrum rather than a dichotomy. We found that enlargement of the INL due to microcysts is not solely responsible for the overall inverse correlation between RGC loss and INL thickening in patients with ON. Even after eyes with microcysts were excluded from the analysis, INL thickness was still significantly larger in ON eyes as compared to normal controls, while its inverse correlation with GCL/IPL thickness was not affected. This suggests that whilst these patients represent a severe form of GCL/IPL loss and INL thickening, they do not necessarily constitute a separate phenotype. In fact, some studies have documented that INL microcysts are not MS-specific, as they have been identified in patients with non-MS ON [15,21], glaucoma [21], neuro-myelitis optica [22,23], Leber’s hereditary optic neuropathy [16], dominant optic neuropathy [16], compressive optic neuropathy [14] and after vitreo-retinal surgery [24]. What all those patients have in common, however, is severe loss of RGCs and their axons.

It is difficult to speculate upon the nature of INL enlargement in ON and the occurrence of microcysts in particular. Various explanations have been put forward, including vitreal traction [16], trans-synaptic degeneration [25], anti-KIR4.1 autoantibodies [11,25], and Muller cell pathology [15,21,23]. However, there have been no longitudinal studies to prove that retrograde degeneration leads to INL changes from proximal lesions such as in the optic nerve. Not all patients with microcysts have been shown to have vitreal tractions [26]. It is possible that non-myelin related primary retinal inflammation with breakdown of the blood-retinal barrier and factors such as microglial activation are responsible for changes in the INL and the development of microcysts [11,13,22]. It does not, however, explain the prevalence of microcysts in ON eyes and the negative association between INL enlargement and GCL/IPL loss. Macula edema has also previously been reported in MS patients in association with subclinical uveitis or retinal periphlebitis [27,28]. However, it is not clear whether these microcysts represent edema. They are not limited to areas with vascular sheathing and fluorescein angiography found no leakage in the eyes of patients with microcysts in patients with glaucoma, hereditary optic neuropathy and post-vitrectomy, making a non-inflammatory explanation more plausible [15,16,21,24]. However in one study, fluorescein leakage was seen in the eyes of one ON patient with binocular microcysts and retinal periphlebitis [11]. It must be noted that the patients included in the study did not all undergo thorough dilated ophthalmic examinations and investigations, so the influence of other comorbidities on the presence of INL microcysts cannot be excluded. Fingolimod is known to be associated with macula edema, however it is difficult to ascertain its influence on retinal layer thickness and microcysts as only 3 patients in our study were on this medication, only one of which had INL microcysts.

In our study, the greatest loss of RNFL and GCL/IPL and thickening of the INL was seen between the optic disc and fovea rather than temporal retina. Among the 5 ON eyes with microcysts, we noted a greater number of cysts in these regions of the retina, a similar finding to another retrospective study on optic neuropathies including glaucoma [21]. Based on such topographical changes, we speculate that the inverse association between RGC loss and INL enlargement could relate to structural changes in the retinal layers. Opposing traction of the tough inner limiting and Bruch’s membranes and/or the supporting role of Muller cells across the thickness of the retina may be partly responsible for maintaining retinal structure, thus resulting in compensatory enlargement of the INL in cases when the RNFL suddenly and severely thins. This is in line with the elongated appearance of the microcysts, which has been reported in other studies [21,24].

Histological and prospective studies are required to test such pathophysiological associations. It is possible that the distribution and prevalence of microcysts may have been under-estimated in our study as areas between the OCT radial lines were not scanned. Our study is also limited as it was cross-sectional and the sample size of patients with INL microcysts was small. In addition, although we attributed changes in the GCL/IPL measurement to RGC neuron loss, we acknowledge that changes in the IPL such as RGC dendrites and amacrine neurons may also have contributed to thickness variability in our study. Furthermore, by reducing inter-eye variability using asymmetry analysis we may have under-estimated the magnitude of changes in the retinal layers given that some RNFL reduction in fellow eyes has been well reported in previous studies.

## Conclusions

Thickening of the INL in MS-related ON correlates with the severity of RGC loss. This is a continuous relationship and the patients with characteristic INL microcysts of the macula may represent the extreme end of the scale, rather than a separate phenotype. Further studies are required to identify what primary or secondary pathophysiological process is responsible for INL changes in MS-related ON.
